# Post-explant profiling of subcellular-scale carbon fiber intracortical electrodes and surrounding neurons enables modeling of recorded electrophysiology

**DOI:** 10.1088/1741-2552/acbf78

**Published:** 2023-03-17

**Authors:** Joseph G Letner, Paras R Patel, Jung-Chien Hsieh, Israel M Smith Flores, Elena della Valle, Logan A Walker, James D Weiland, Cynthia A Chestek, Dawen Cai

**Affiliations:** 1 Department of Biomedical Engineering, University of Michigan, Ann Arbor, MI 48109, United States of America; 2 Biointerfaces Institute, University of Michigan, Ann Arbor, MI 48109, United States of America; 3 Neuroscience Graduate Program, University of Michigan, Ann Arbor, MI 48109, United States of America; 4 Department of Electrical Engineering and Computer Science, University of Michigan, Ann Arbor, MI 48109, United States of America; 5 Biophysics Program, University of Michigan, Ann Arbor, MI 48109, United States of America; 6 Department of Computational Medicine and Bioinformatics, Michigan Medicine, Ann Arbor, MI 48109, United States of America; 7 Department of Ophthalmology and Visual Sciences, Kellogg Eye Center, University of Michigan, Ann Arbor, MI 48105, United States of America; 8 Robotics Department, University of Michigan, Ann Arbor, MI 48109, United States of America; 9 Department of Cell and Developmental Biology, University of Michigan Medical School, Ann Arbor, MI 48109, United States of America

**Keywords:** electrophysiology, neural probes, immunohistochemistry, motor cortex, neuron density, electrophysiological modeling, intracortical electrodes

## Abstract

*Objective.* Characterizing the relationship between neuron spiking and the signals that electrodes record is vital to defining the neural circuits driving brain function and informing clinical brain-machine interface design. However, high electrode biocompatibility and precisely localizing neurons around the electrodes are critical to defining this relationship. *Approach.* Here, we demonstrate consistent localization of the recording site tips of subcellular-scale (6.8 *µ*m diameter) carbon fiber electrodes and the positions of surrounding neurons. We implanted male rats with carbon fiber electrode arrays for 6 or 12+ weeks targeting layer V motor cortex. After explanting the arrays, we immunostained the implant site and localized putative recording site tips with subcellular-cellular resolution. We then 3D segmented neuron somata within a 50 *µ*m radius from implanted tips to measure neuron positions and health and compare to healthy cortex with symmetric stereotaxic coordinates. *Main results.* Immunostaining of astrocyte, microglia, and neuron markers confirmed that overall tissue health was indicative of high biocompatibility near the tips. While neurons near implanted carbon fibers were stretched, their number and distribution were similar to hypothetical fibers placed in healthy contralateral brain. Such similar neuron distributions suggest that these minimally invasive electrodes demonstrate the potential to sample naturalistic neural populations. This motivated the prediction of spikes produced by nearby neurons using a simple point source model fit using recorded electrophysiology and the mean positions of the nearest neurons observed in histology. Comparing spike amplitudes suggests that the radius at which single units can be distinguished from others is near the fourth closest neuron (30.7 ± 4.6 *µ*m, $\bar{\text{X}}$ ± S) in layer V motor cortex. *Significance.* Collectively, these data and simulations provide the first direct evidence that neuron placement in the immediate vicinity of the recording site influences how many spike clusters can be reliably identified by spike sorting.

## 1. Introduction

Recording and interpreting neural activity in the mammalian cortex is paramount to understanding brain function and for controlling brain-machine interfaces (BMIs). Capturing the signals of individual neurons yields the most fundamental dynamics of neural activity (Harris *et al* 2016, Hong and Lieber 2019), thereby providing the highest precision when decoding neural circuits (Schwartz *et al* 2006). Intracortical electrode recordings have high spatiotemporal resolution to capture individual neurons’ signaling associated with fast-paced behaviors (Chorev *et al* 2009). Therefore, electrode architectures with many dense recording sites are desired for sampling large populations of neurons (Seymour *et al* 2017, Hong and Lieber 2019).

Currently, the most widely used intracortical electrodes are composed of silicon shanks fabricated using standard cleanroom techniques (HajjHassan *et al* 2008). These electrodes are becoming increasingly sophisticated, with newer designs approaching or exceeding a thousand densely-packed recording sites (Shobe *et al* 2015, Scholvin *et al* 2016, Jun *et al* 2017, Steinmetz *et al* 2021, Zardini *et al* 2021). For example, the Neuropixels probe has demonstrated simultaneous recording from hundreds of individual neurons along its length (Jun *et al* 2017, Steinmetz *et al* 2021, Paulk *et al* 2022). Planar silicon electrode arrays, e.g. the Utah Electrode Array (UEA), can sample wide areas of cortex (Nordhausen *et al* 1996) for use in BMIs (Serruya *et al* 2002), enabling the restoration of function lost to neurological disease (Hochberg *et al* 2012, Collinger *et al* 2013, Pandarinath *et al* 2017). Moreover, the recent advancements of multi-shank Michigan-style electrodes (Scholvin *et al* 2016), such as the Neuropixels 2.0 (Steinmetz *et al* 2021), and variable-length UEA-style electrodes, such as the Sea of Electrodes Array (Zardini *et al* 2021), signify that recording neuronal populations in 3D is possible.

Extensive evidence, however, indicates that chronic implantation of silicon electrodes can elicit a multi-faceted foreign body response (FBR) at the implant site, which includes significant glial scarring (Turner *et al* 1999), high microglia presence (Szarowski *et al* 2003), large voids of tissue (Nolta *et al* 2015, Black *et al* 2018), blood-brain-barrier disruption (Saxena *et al* 2013), and neurodegeneration (Biran *et al* 2005, Winslow *et al* 2010). Recent studies have uncovered more effects, including hypoxia and progressive neurite degeneration (Welle *et al* 2020a), a shift toward inhibitory activity (Salatino *et al* 2017b), myelin injury and oligodendrocytes loss (Chen *et al* 2021), and mechanical distortion of neurons (Du *et al* 2017, Eles *et al* 2018). RNA sequencing of implant site tissue also yielded differential expression of more than 100 genes, signifying the FBR results in complex biological changes (Thompson *et al* 2021). Moreover, recorded signal amplitudes degrade over chronic time periods (Chestek *et al* 2011, Sponheim *et al* 2021) and can both increase and decrease during experimental sessions (Chestek *et al* 2011, Perge *et al* 2013), which have been attributed to the FBR (Nolta *et al* 2015). The chronic neuron loss, particularly within the single unit recording range (Henze *et al* 2000, Buzsáki 2004), brings to question silicon electrodes’ ability to reliably record activity attributed to individual neurons chronically. Silicon probes’ large size has been implicated as a primary reason for the FBR (Szarowski *et al* 2003, Seymour and Kipke 2007, Thompson *et al* 2020). Many newer electrode technologies have been designed to overcome the FBR (Salatino *et al* 2017a, Hong and Lieber 2019, Thompson *et al* 2020). In particular, smaller electrodes that have cellular to subcellular dimensions (Kozai *et al* 2012, Luan *et al* 2017, Deku *et al* 2018, Musk 2019, Yang *et al* 2019) generate smaller tissue displacement (Obaid *et al* 2020a) and reduced FBR and neuron loss (Seymour and Kipke 2007, Thompson *et al* 2020), suggesting an ability to record more naturalistic neural populations.

While computational models have been proposed to decode the relationship between recorded spikes and contributing neurons (Moffitt and McIntyre 2005, Pettersen and Einevoll 2008, Lempka *et al* 2011, Mechler and Victor 2012, Malaga *et al* 2015), their validity remains unresolved without empirical measurements of neuron locations and their spike timing (Marques-Smith *et al* 2020). Attempts at acquiring these ‘ground truth’ measurements have been performed using tetrodes or electrodes with similar geometry (Henze *et al* 2000, Du *et al* 2009, Mechler and Victor 2012), which Marques-Smith *et al* (2020) assert as differing considerably from state-of-the-art electrodes such as Neuropixels (Jun *et al* 2017), under *ex vivo* conditions (Anastassiou *et al* 2015, Yger *et al* 2018), simulated data (Pedreira *et al* 2012, Magland *et al* 2020, Buccino and Einevoll 2021), or acutely *in vivo* (Neto *et al* 2016, Allen *et al* 2018) and with low resolution in cell localization (Marques-Smith *et al* 2020). As modeling suggests that biofouling in the electrode-tissue interface influences neural recording quality (Malaga *et al* 2015), ‘ground-truth’ must be measured in cases where electrodes are implanted *in vivo* over long time periods. Precisely localizing chronically implanted electrodes *in situ* and surrounding neurons has begun to bridge this gap (Luan *et al* 2017, Yang *et al* 2019, Patel *et al* 2020, Sharon *et al* 2021).

Subcellular-scale (6.8 *µ*m diameter) carbon fiber electrodes that elicit a minimal FBR and maintain high neuron densities after chronic implantation (Kozai *et al* 2012, Patel *et al* 2016, 2020, Welle *et al* 2020b) are ideal candidates for acquiring these ‘ground truth’ recordings. Previously, we demonstrated a ‘slice-in-place’ technique to retain the electrodes in brain slices for localizing the recording tips in deep brain structures (Patel *et al* 2020). However, brain curvature and the bone screws required for skull-mounted headcaps render this method incompatible with fibers implanted in shallower cortical regions. In this report, we demonstrate that explanting carbon fiber electrodes from cortical recording sites followed by slicing thick horizontal brain sections with headcaps removed retains the ability to localize the tips with high-resolution 3D images. We used rats chronically implanted in layer V motor cortex to assess neuronal health and glial responses and for modeling the relationship between recorded spikes and surrounding neurons. From 3D reconstructions of neuron somas, we found a minimal loss of 18% in mean neuron count per volume, although neurons were stretched compared to neurons in the contralateral hemisphere. The distance of the nearest neuron to implanted fibers (17.2 ± 4.6 *µ*m, $\bar{\text{X}}$ ± S) was close to that of simulated electrodes positioned in the contralateral hemisphere (16.2 ± 4.8 *µ*m, $\bar{\text{X}}$ ± S) such that the distances were not significantly different. Given the minimal disruption in surrounding neurons, we modeled the extracellular spikes that could be recorded from the neuron population at the implant site, which suggested that their natural distribution is a fundamental limiting factor in the number of spike clusters that can be sorted.

## 2. Methods

### 2.1. Carbon fiber electrode array fabrication

High density carbon fiber (HDCF) electrode arrays with 16 channels were fabricated using previously reported methods (Huan *et al* 2021). Briefly, silicon support tines were fabricated from 4” silicon wafers using silicon micromachining processes. The support tines had trenches etched into them via deep reactive ion etching to hold the fibers for facile insertion into the brain, tapered to a width of 15.5 *µ*m, had a pitch of 80 *µ*m, and had a length of 3 mm for targeting cortex. The support tines had gold pads on them to interface with the carbon fibers. These gold pads then led to a separate set of gold bond pads to interface with a printed circuit board (PCB). Once tines were fabricated, they were bonded to a custom PCB with Epo-Tek 301 epoxy (Epoxy Technology, Billerica, MA). 2-Part epoxy (1FBG8, Grainger, Lake Forest, IL) was applied to the underside of the silicon portion to provide buttress support. The gold bond pads were then wire-bonded to pads on the PCB and the wire bonds coated in Epo-Tek 353ND-T epoxy. Carbon fibers were then laid into the support tines by exploiting capillary action from a combination of deionized water (electrical pad end) and Norland Optical Adhesive 61 (NOA 61) (Norland Products, Inc., Cranbury, NJ) (distal end). A NLP 2000 (Advanced Creative Solutions, Carlsbad, CA) was used to apply Epo-Tek H20E silver epoxy to the gold pads and the carbon fibers to establish an electrical connection. NOA 61 was applied to the gold pads and the carbon fibers to further secure them. Fibers were then cut to a length of 1000 *µ*m and coated with ∼800 nm of Parylene C (PDS2035CR, Specialty Coatings Systems, Indianapolis, IN). Fibers were then laser cut to a final length of 300 *µ*m beyond the silicon support tine ends with a 532 nm Nd:YAG pulsed laser (LCS-1, New Wave Research, Fremont, CA) as described previously (Welle *et al* 2020b). Carbon fibers were then plasma ashed in a Glen 1000P Plasma Cleaner (Glen Technologies Inc., Fremont, CA). Fiber tips were functionalized in one of two ways: (1) electrodeposition by dipping carbon fibers in a solution of 0.01 M 3,4-ethylenedioxythiophene (483 028, MilliporeSigma, Darmstadt, Germany) and 0.1 M sodium p-toluenesulfonate (152 536, MilliporeSigma) and applying 600 pA/channel using a PGSTAT12 potentiostat (EcoChemie, Utrecht, Netherlands) to coat the tips with PEDOT:pTS (Patel *et al* 2016, Welle *et al* 2020b) (*N* = 3 arrays) or (2) electrodeposition of Platinum Iridium (PtIr) with a Gamry 600+ potentiostat (Gamry Instruments, Warminster, PA) (*N* = 2 arrays) using previously published methods (della Valle *et al* 2021). Silver ground and reference wires (AGT05100, World Precision Instruments, Sarasota, FL) were soldered to the PCB, completing assembly. For one electrode, a support tine was broken off prior to implant as a sham channel for a separate study (for rat #1). Once electrodes were completed, electrochemical impedance spectroscopy (EIS) was measured with electrodes immersed in 1x phosphate buffered saline (PBS) using previously published methods (Kozai *et al* 2012, Patel *et al* 2015). Impedances at 1 kHz were measured to be 129.4 ± 259.0 kΩ ($\bar{\text{X}}$ ± S) (*n* = 79 fibers, five electrode arrays), where probes functionalized with PEDOT:pTS were measured at 24.6 ± 20.8 kΩ (*n* = 47 fibers, three electrode arrays) and probes functionalized with PtIr were measured at 283.4 ± 353.7 kΩ (*n* = 32 fibers, two electrode arrays). All electrodes underwent ethylene oxide gas sterilization prior to implantation.

### 2.2. Electrode implantation

Adult male Long-Evans rats (*N* = 5) weighing 393–630 g were implanted with one HDCF electrode array each. Surgical implantation closely followed previously reported procedures (Patel *et al* 2015, Welle *et al* 2020b). Throughout the surgeries, temperature was monitored with a rectal thermometer and breath rate was monitored with a pulse oximeter. Isoflurane (5% (v/v) induction, 1%–3% maintenance) was used as a general anesthetic and carprofen (5 mg kg^−1^) as a general analgesic. After opening the scalp, seven bone screws (19010-00, Fine Science Tools, Foster City, CA) were screwed into the skull. One screw at the posterior end of the skull was used for referencing. A 2 × 2 mm craniotomy was drilled in the right hemisphere, where the bottom left corner of the craniotomy was 1 mm lateral and 1 mm anterior to bregma. The probe was then lowered to the dura mater to zero its dorsal/ventral position. After durotomy with a 23 G needle, the probe was immediately inserted to a depth of 1.2–1.3 mm to reach layer V of motor cortex. The craniotomy was then filled with DOWSIL silicone gel (DOWSIL 3-4680, Dow Silicones Corporation, Midland, MI). Ground and reference wires were wrapped around the most posterior bone screw for referencing. A headcap was formed by applying methyl methacrylate (Teets denture material, 525 000 & 52 600, Co-oral-ite Dental MFG. Co., Diamond Springs, CA) onto the skull until the probe’s electrical connector was firmly in place and bone screws were covered. The scalp was sutured around the connector and surgery was complete.

It is important to note that rat #2 was one of the rats reported in Welle *et al* (2020b), but only up through day 63 of 92 and with a focus on electrophysiological yield over time.

### 2.3. Electrophysiological recording and spike sorting

Electrophysiological recordings were collected in chronically implanted rats while awake and freely moving in a Faraday cage (Welle *et al* 2020b). Signals were recorded using ZC16 and ZC32 headstages, RA16PA pre-amplifiers, and a RX7 Pentusa base station (Tucker-Davis Technologies, Alachua, FL) at 24 414.1 Hz. Recordings were collected at least weekly for 10 min sessions. Spike sorting was semi-automated and based upon previously reported procedures (Patel *et al* 2016, Welle *et al* 2020b). Channels were excluded from a session if the impedance at 10 Hz was abnormally high compared to other channels and previous sessions (∼1–2 weeks), where impedance was measured using EIS (Patel *et al* 2016). This exclusion was based on exclusion criteria reported in Patel *et al* (2016). However, no channels were excluded if 10 Hz impedances were not collected or measured using different methods (rats 1 & 3). Common average referencing was performed using the remaining channels to reduce noise (Ludwig *et al* 2009). The following steps were performed in Plexon Offline Sorter (version 3.3.5) (Plexon Inc., Dallas, TX) by a trained operator. Signals were high-pass filtered using a 250 Hz four-pole Butterworth filter. Five 100 ms snippets of signal with low neural activity and artifact noise were manually selected from each channel and used to measure *V*_RMS_ noise for each channel (Patel *et al* 2016). The threshold for each channel was set at −3.5 × *V*_RMS_. Cross channel artifacts were then invalidated. Putative cluster centers were manually designated and waveforms assigned using K-Means clustering. Obvious noise waveform clusters were removed. Automated clustering was performed using the Standard Expectation-Maximization Scan function in Plexon Offline Sorter (Welle *et al* 2020b). Persisting noise waveforms were removed, and obvious oversorting and undersorting errors were manually corrected. Clusters were also cleaned manually. Resultant waveforms were imported into and analyzed in MATLAB (version R2020b) (MathWorks, Natick, MA) using custom scripts. Electrophysiological recording capacity at the experimental endpoints for each probe are shown in figure S1.

### 2.4. Tissue preparation, immunohistochemistry, and imaging

At the end of the implantation period, rat brains were prepared for immunohistochemistry and histological imaging. Rats were transcardially perfused on day 88–92 (*N* = 3) or day 42 (*N* = 2) as described previously (Patel *et al* 2016, Welle *et al* 2020b). If the perfusion fixation was successful, brains were extracted and soaked in 4% paraformaldehyde (PFA) (19 210, Electron Microscope Sciences, Hatfield, PA) in 1x PBS for 1–3 d. If more fixation was required, brains remained in the skull while soaking in PFA solution for two days before extraction, followed by an additional 24-hour incubation in PFA solution. In all cases, the electrode array, headcap, and skull-mounted bone screws were removed from the brain. Brains were then incubated in 30% sucrose (S0389, MilliporeSigma) in 1x PBS with 0.02% sodium azide (S2002, MilliporeSigma) for at least 72 h until cryoprotected. Brains were then sliced to a thickness of 300 *µ*m (Patel *et al* 2020) with a cryostat. Slices were selected for staining based upon estimated depth and/or from the observation of holes in a brightfield microscope.

Immunohistochemistry closely followed previously reported staining techniques (Welle *et al* 2020b), but modified to accommodate 300 *µ*m brain slices (Patel *et al* 2020). All incubation periods and washes were performed with brain slices in well plates on nutators. Chosen slices were first incubated in 4% PFA (sc-281 692, Santa Cruz Biotechnology, Dallas TX) for 1 d at 4 °C. Slices were washed for 1 h in 1x PBS twice at room temperature and then incubated in a solution containing 1% Triton X-100 (93 443, MilliporeSigma) in StartingBlock (PBS) Blocking Buffer (37 538, ThermoFisher Scientific, Waltham, MA) overnight at room temperature to permeabilize and block the tissue, respectively. Slices were then washed in 0.1%–0.5% Triton X-100 in 1x PBS (PBST) solution for one hour at room temperature three times.

Slices were incubated in primary antibodies for 7 d at 4 °C, where antibodies were added to a solution containing 1% Triton X-100, 0.02% sodium azide (1% of solution containing 2% sodium azide in 1x PBS), and StartingBlock. The primary antibody cocktail differed between rats implanted for 6 weeks (*N* = 2) and 12+ weeks (*N* = 3). In all rats, antibodies staining for neurons (Mouse anti-NeuN, MAB377, MiliporeSigma) and astrocytes (Rabbit anti-Glial fibrillary acidic protein (GFAP), Z0334, Dako/Agilent, Santa Clara, CA) were used, where both had dilution ratios of 1:250. For 6 week rats, staining for axon initial segments (AIS) with Goat anti-Ankyrin-G (1:1000 dilution ratio) was added. The Ankyrin-G antibody was made and provided by the Paul Jenkins Laboratory (University of Michigan, Ann Arbor, MI) using methods published previously (He *et al* 2014). For 12+ week rats, staining for microglia (Guinea Pig anti-IBA1, 234 004, Synaptic Systems, Göttingen, Germany) (1:250 dilution ratio) was added. Slices were washed in 0.1%–0.5% PBST three times at room temperature for one hour each wash before secondary antibody incubation at 4 °C for 5 d. Secondary antibodies used the same base solution as the primary cocktail. The following secondary antibodies were used: Donkey anti-Mouse Alexa Fluor 647 (715-605-150, Jackson ImmunoResearch Laboratories, Inc., West Grove, PA) for neurons, Donkey anti-Rabbit Alexa Fluor 546 (A10040, Invitrogen, Carlsbad, CA) for astrocytes in 6 week rats, Goat anti-Rabbit Alexa Fluor 532 (A11009, Invitrogen) for astrocytes in 12+ week rats, Donkey anti-Guinea Pig Alexa Fluor 488 (706-545-148, Jackson ImmunoResearch Laboratories, Inc.) for microglia, Donkey anti-Goat-Alexa Fluor 488 (705-545-003, Jackson ImmunoResearch Laboratories, Inc.) for AISs, which all had dilution ratios of 1:250, and 4ʹ,6-Diamidino-2-Phenylindole, Dihydrochloride (DAPI) (D1306, Invitrogen) for cellular nuclei (1:250–1:500 dilution ratio). Slices were washed in 0.1%–0.5% PBST twice at room temperature for two hours each wash and were washed in 1x PBS with 0.02% azide for at least overnight before imaging could commence. Stained slices were stored in 1x PBS with 0.02% azide at 4 °C outside of imaging sessions after staining.

Prior to imaging, slices were rapidly cleared using an ultrafast optical clearing method (FOCM) (Zhu *et al* 2019). FOCM was prepared as a solution of 30% (w/v) urea (BP169500, ThermoFisher Scientific), 20% (w/v) D-sorbitol (DSS23080-500, Dot Scientific, Burton, MI), and 5% glycerol (w/v) (BP229-1, ThermoFisher Scientific) in dimethyl sulfoxide (DMSO) (D128-500, ThermoFisher Scientific). Glycerol was added at least one day after other reagents started mixing and after urea and D-sorbitol were sufficiently dissolved in DMSO. FOCM was diluted in either MilliQ water (*N* = 1 rat) or 1x PBS (*N* = 4 rats) to 25%, 50% and 75% (v/v) solutions. During clearing, slices were titrated to 75% FOCM in 25% concentration increments, 5 min per step. 75% FOCM in water was found to expand tissue laterally (6.7%) after imaging rat #2, so other slices from other rats were cleared with FOCM in 1x PBS. This expansion was not corrected in subsequent analyses. The clearing process was repeated immediately before each imaging session. The samples remained suspended in the 75% FOCM solution during imaging. Images were collected with a Zeiss LSM 780 Confocal Microscope (Carl Zeiss AG, Oberkochen, Germany) with 10x and 20x objectives. The microscope recorded transmitted light and excitation from the following lasers: 405, 488, 543, and 633 nm. Z-stacks of regions of interested were collected along most, if not all, of the thickness of the sample. Images had XY pixel size of 0.81 *µ*m or 0.202 *µ*m and z-step of 3 *µ*m or 0.5–0.6 *µ*m, respectively. Laser power and gain were adjusted to yield the highest signal-to-noise (SNR) ratio while also minimizing photobleaching. The ‘Auto Z Brightness Correction’ feature in ZEN Black (Carl Zeiss) was used to account for differences in staining brightness along the slice thickness. The implant site was located by overview imaging and quick scanning, where high astrocyte staining signal in dorsal regions and a straight line of holes delimited the electrode site. Sites with approximately similar stereotaxic coordinates to those of the implant site but in the contralateral hemisphere of the same slice were imaged as control. After imaging, slices were titrated back to 0.02% sodium azide in 1x PBS, and stored. Clearing was repeated again when further imaging was needed, as each implant site required multiple imaging sessions.

### 2.5. Putative tip localization

Putative carbon fiber electrode tips were localized independently by three reviewers experienced in histology to estimate the confidence and the reproducibility of localization. Tips were localized using ImageJ (Fiji distribution, Schindelin *et al* 2012) with additional cross-referencing in Zen Black (2012) (Zeiss Microscopy). Fiber tips were localized by first identifying electrode tracts in more dorsal focal planes. These tracts presented as dark holes in transmitted light and fluorescent imaging channels that were typically positioned in an approximately straight line and surrounded by high GFAP intensities. Putative electrode tracts were enhanced by using a combination of basic imaging processing and visualization techniques including contrast adjustment, histogram matching (Miura 2020) to account for changes in brightness throughout the brain slice, median or Gaussian filters (including 3D versions (Ollion *et al* 2013)) for reducing noise, maximum intensity projections, and toggling the imaging channels that were visualized simultaneously. The estimated tip location was determined by finding the *z*-plane and *x-y* coordinates at which the tract rapidly began to reduce in size and become filled with surrounding background fluorescence or parenchyma when scrolling dorsal-ventrally through the image. The tip width, height, and center were determined by manually fitting an ellipse to the electrode tract.

### 2.6. Verification of tip localization

Putative tip localization was verified by measuring electrode tract pitches, tip cross-sectional area, and the agreement between three reviewers in tip localization. Electrode tract pitches were measured in ImageJ after stitching images (Preibisch *et al* 2009). The positions of electrode tracts were measured in the same z-step or in nearby *z*-steps to achieve an approximately planar arrangement of tracts. Ellipses were manually fit to tracts, and the Euclidean distance between the centers of neighboring tracts were measured in MATLAB to determine pitch. The cross-sectional area of each tip was calculated as the area of the ellipse fit to the tip localized as described above. The agreement between three reviewers experienced in histology was determined by measuring the absolute value of the Euclidean distance between the three centers of the ellipses fit to each fiber. When determining the localization confidence, all comparisons for each group of fibers were grouped together. The median and interquartile range were measured from each group of comparisons.

Fiber tips that were not localized by all three reviewers were excluded from further analyses and from agreement determination. Three fibers were excluded from rat #5 because one fiber was not found by one reviewer, and two fibers were found in different images than the other reviewers, so localizations were not directly comparable. Tissue damage in rat #3 was so severe (figure S3) that even the number of fibers in the tissue was not consistently identified by reviewers, therefore rat #3 was excluded from quantitative analyses. For cross-sectional area measurements and subsequent analyses requiring fiber localization, the positions localized by the first author were used, where exclusion from other reviewers were not considered.

### 2.7. Segmenting neuron somas into 3D scaffolds

The somas of neurons surrounding electrodes were manually segmented in 3D after putative fiber localization to measure soma morphology and determine the Euclidean distance between neuron somata centroids and carbon fiber electrode tips, which we called the neurons’ relative positions. We intentionally segmented a larger number of neurons than required, as neuron proximity to putative fibers is difficult to determine from volumetric confocal imaging by inspection alone. Therefore, we segmented any neuron that was wholly or partially present within the volume of a 100 × 100 *µ*m (diameter *X* height) cylinder centered at each electrode tip to ensure that any neuron with a centroid within a 50 *µ*m radius from the fiber tips would be segmented and computer algorithms could determine measurements onward (e.g. relative distance, morphometrics). Image pre-processing and segmentation were performed using ImageJ plugins. The NeuN channel was used to visualize neuron somas for segmentation. First, a *z*-step with a qualitatively high SNR was selected and contrast adjusted to make the image clearer. The remaining *z*-steps in the *z*-stack were histogram matched (Miura 2020) to the selected *z*-step to account for variations in brightness throughout the slice along its thickness. A 3D median filter (Ollion *et al* 2013) was applied to reduce noise. These image pre-processing steps ensured that the somata, stained by NeuN, had high SNRs and were clearly differentiable from other tissue. The ImageJ plugin nTracer (primarily version 1.3.5) (Roossien *et al* 2019) was used to segment each neuron by manually tracing along the cell outline in each *z*-step that the neuron was present. Neurons were also traced in contralateral regions as a control. Figure S2 shows several *z*-planes of two neurons that were manually traced and a single plane that segmented many neurons to provide better illustration. For rat #1, five points with similar coordinates to fiber tip centroids were selected and neurons traced within a cylinder (100 *µ*m diameter, 100 *µ*m height) (*N* = 5 hypothetical fibers, *N* = 199 neurons). For rat #2, all neurons that bounded voxels in a cylindrical region (300 *µ*m diameter, 300 *µ*m height) were traced (*N* = 926 neurons). Traced neurons were imported into MATLAB using custom scripts.

For some fibers (rat #1: *N* = 8 fibers; rat #2: *N* = 5 fibers), the electrode tip was closer to the top of the brain section than 50 *µ*m. Instead of 50 *µ*m, the distance between the fiber tip and the top of the brain section at that fiber’s tract was selected as half the cylinder height for tracing and used in subsequent analyses as the radius around that fiber. Additionally, neuron somas that were cut along the plane of cryosectioning were excluded if it was clear that the middle of the soma was excluded from the brain slice.

### 2.8. Neuron morphometrics and densities

Neuron morphometrics and positions were extracted from the 3D scaffolds of neurons produced by tracing. These measurements were performed in MATLAB using custom scripts after having been imported into MATLAB storage formats. To measure neuron soma volume, all voxels bounded by the scaffold were counted and the total number was multiplied by the volume of a single voxel (Prakash *et al* 1993). The centroids of these sets of points, which were 3D point clouds, were used to determine neuron positions and distances relative to the putative electrode tips. Centroid positions were also used for counting the number of neurons within 3D radii, such as 50 *µ*m, and in determining neuron density. To determine the extent of neuron elongation around implanted fibers, the cell shape strain index (CSSI) as defined by Du *et al* (2017) with equation (1) was measured. An ellipse was fit to the soma trace in the *z*-step that had the greatest cross-sectional area for that neuron (Ohad Gal. fit_ellipse [www.mathworks.com/matlabcentral/fileexchange/3215-fit_ellipse], MATLAB Central File Exchange). CSSI was determined using equation (1) (Du *et al* 2017), where *a* is the minor axis and *b* is the major axis from elliptical fitting:

\begin{equation*}CSSI = \frac{{b - a}}{{\frac{{b + a}}{2}}}.\end{equation*}

To measure the length of neurons in the dorsoventral direction parallel to the axis of explantation, the number of *z*-steps in which each neuron was traced was multiplied by the image’s *z*-step resolution.

These same measurements were repeated for neurons in contralateral sites. Comparisons of neurons within a 50 *µ*m spherical radius of localized tips included neurons where the centroid was within 50 *µ*m. To determine the relationship between distance and CSSI or volume, only neurons that were ventral to all tips analyzed in the source image were included to remove the effects of multiple nearby tips.

### 2.9. Nearest neuron positions

Neuron positions relative to implanted fibers were determined by calculating the Euclidean distance between the center of the ellipse manually fitted to each carbon fiber electrode tip (see *Tip localization*) and centroid of the point cloud bounded by each traced neuron’s 3D scaffold. Therefore, the neuron’s position was the centroid of all points bounded by the NeuN stain. These distances were sorted from shortest to longest to determine the nearest neuron positions relative to implanted fibers. When determining the mean neuron position, if that position was not measured for one or more fibers due to a reduced tracing radius, that fiber was excluded from the mean neuron position calculation for that position.

Similar measurements were performed at contralateral sites as a control. In *N* = 1 rat, neurons within 50 *µ*m of five hypothetical fiber points with similar coordinates to localized fiber tips were traced, and the nearest neuron positions were determined in the same manner as with implanted fibers. For another rat, all neurons within a 300 × 300 *µ*m (diameter *X* height) cylindrical volume were traced. Points were placed in a 3D grid with 12.5 *µ*m spacing within this volume but were excluded if positioned within 50 *µ*m of the volume’s border or if within the set of points bounded by a traced neuron. These points were used as hypothetical carbon fiber tip locations in unimplanted contralateral tissue. Similarly to implanted fibers, the Euclidean distance between each hypothetical point and the centroid of all points bounded by each traced neuron was measured to determine relative neuron distances. As described earlier (see *Neuron morphometrics and densities*), these neuron centroid positions were also used to determine the neuron densities and neuron counts within spherical radii from hypothetical fiber locations.

### 2.10. Glial responses around implanted carbon fibers

Glial responses to chronic implantation of carbon fiber electrodes were evaluated by measuring the staining intensity of GFAP and IBA1 surrounding implanted fibers, similar to measurements commonly made previously (e.g. Patel *et al* 2016 and Jang *et al* 2021). *Z*-steps that captured focal planes that included regions of missing tissue due to capturing either the top or bottom of the brain slice were excluded. Fibers were excluded if their tips were localized in excluded *z*-steps. Stitched (Preibisch *et al* 2009) confocal images were imported into MATLAB using the Bio-Formats MATLAB Toolbox (The Open Microscopy Environment, www.openmicroscopy.org/bio-formats/downloads/) for analysis. The background intensity for each *z*-step was determined first. The Euclidean distance of each pixel in the *z*-step to every tip center was determined. If the *z*-step was dorsal to the tip, then the distance to the center of the electrode tract in that *z*-step was determined instead. The mean intensity of pixels that were 300–310 *µ*m away from those tip positions was measured as the background intensity for that *z*-step. Next, a line (the electrode axis) was fit to the coplanar fiber tip positions. The minimum pitch of the electrode tip locations along this line was used to determine bounded lanes centered at each tip for measurement. The intensity of glial fluorescence at increasing distances from each electrode tip were measured by determining the mean intensity of pixels bounded by concentric rings that were 10 *µ*m thick, centered at each electrode tip location, and were bounded by these lanes with equal width to prevent overlap between fibers. Glial intensity was reported as the ratio of the mean intensity for each bin to the mean intensity of the background for the *z*-step containing the tip. Since fiber tips were localized at a range of *z*-steps, the measurement was performed at the appropriate *z*-step for each fiber.

This process was performed for both GFAP and IBA1. As an additional control, the process was repeated using images collected of sites in the same brain sections as the implanted tips with similar stereotaxic coordinates in the contralateral hemisphere.

### 2.11. Modeling of electrophysiology

A simple point source model (Lee *et al* 2007), similar to the model reported in Nason *et al* (2020), was used to predict the extracellular spike waveforms recorded by carbon fiber electrodes that originated from neurons surrounding implanted carbon fibers. This model is defined by equation (2):

\begin{equation*}{V_{{\text{pp}}}}\left( r \right) = \frac{{{I_{{\text{pp}}}}}}{{4\sigma {\pi}{r}}}\end{equation*}

where *V*_pp_ is the extracellular potential recorded by an electrode, *I*_pp_ is the peak–peak extracellular current generated by a neuron firing an action potential, *σ* is the conductivity of the brain tissue between the electrode and the neuron, and *r* is the relative distance between the neuron and the electrode. The model operates on the following assumptions: (1) that the brain is an isotropic medium with regard to frequency (Logothetis *et al* 2007), (2) neurons are treated as point sources (Holt and Koch 1999, Lee *et al* 2007, Nason *et al* 2020), where the point used in this study is the centroid of the NeuN stain (the soma), 3) cell spiking output over time is constant (Jog *et al* 2002) and across neurons (Lempka *et al* 2011, Nason *et al* 2020). Using values for *I*_pp_ and *σ* that were empirically fit or identified in literature renders the equation a single-parameter model, where the relative distance of the source neuron to the electrode, *r*, is the required input.

The model was fit empirically using three methods. In the first, *σ* = 0.27 S m^−1^ was selected from literature (Slutzky *et al* 2010) and *I*_pp_ was determined from fitting. Electrophysiology from rat #2 was used for fitting because all fibers could be putatively localized with high confidence. Also, the penultimate recording session proved to be exemplary, and using recording sessions collected towards the end of the implantation period increases the likelihood of coherence between recorded electrophysiology and histological outcomes (Michelson *et al* 2018). Spike clusters that were recorded 84 d post implant in rat #2 were sorted and ranked by mean amplitude in descending order. Spikes associated with the largest cluster recorded on each channel that recorded sortable units (*N* = 12 channels) were grouped, and the mean peak–peak amplitude was plotted against the mean position of the closest neuron (*N* = 2 rats). This was repeated for the second and third largest clusters and matched to the second and third closest neuron positions, respectively. *I*_pp_ was then fit to these three value pairs using the MATLAB function *lsqcurvefit*. In the second and third methods, the fit was performed using individual spike cluster and neuron distance pairs. For each channel, spike clusters were sorted in descending order by mean peak–peak waveform amplitude. These cluster amplitudes were plotted against the positions of neurons surrounding those channels with the same rank in position (e.g. the amplitude of the second largest cluster plotted against the position of the second closest neuron). One cluster was excluded because the position of the third closest neuron was not measured due to the electrode tip’s proximity to the top of the brain slice. In the second method, both *σ* and *I*_pp_ were fit using *lsqcurvefit*. In the third method, *σ* = 0.27 S m^−1^ and *I*_pp_ was fit.

To plot predicted waveforms, all spikes sorted across all clusters on day 84 for rat #2 were collected and normalized to have peak–peak amplitudes of 1. The mean positions of the nearest ten neurons were used as input into equation (2) using the first empirical fit, which yielded the scaling factor for the normalized spikes. The mean and standard deviation of the scaled spikes was evaluated for each position and plotted in figures 5(d) and S8.

### 2.12. Statistical analyses

Comparisons of neuron soma volumes and CSSIs between the neurons surrounding implanted fibers and control regions were performed using two-sided Kolmogorov–Smirnov tests (Du *et al* 2017) with an alpha of 0.05. Neuron densities, neuron positions, and glial intensities in radial bins surrounding the implanted fibers compared to control were performed using two-sided two sample *t*-tests with an alpha of 0.05. Linear regressions were determined using the *fitlm* function in MATLAB. All statistical tests were performed in MATLAB.

### 2.13. Figures and graphics

All figures were generated using a combination of ImageJ, MATLAB (both versions R2020b and R2022a), Inkscape (version 1.1.2), and Adobe Illustrator 2022 (version 26.1). ImageJ was used for figures showing histology. MATLAB produced numerical plots. Inkscape was used to compile and complete these figures, with some help from Adobe Illustrator. Videos were produced using ImageJ and Adobe Media Encoder 2022 (22.61).

### 2.14. MATLAB code add-ons

In addition to previously stated code add-ons for MATLAB, we used the following: shadederrorbar (Rob Campbell. raacampbell/shadedErrorBar (https://github.com/raacampbell/shadedErrorBar), GitHub) (multiple versions) and subtightplot (Felipe G. Nievinski. subtightplot (www.mathworks.com/matlabcentral/fileexchange/39664-subtightplot), MATLAB Central File Exchange.). Both were used for figure generation.

## 3. Results

### 3.1. Assessment of FBRs induced by whole carbon fiber electrode arrays implanted chronically

**Figure 1.**
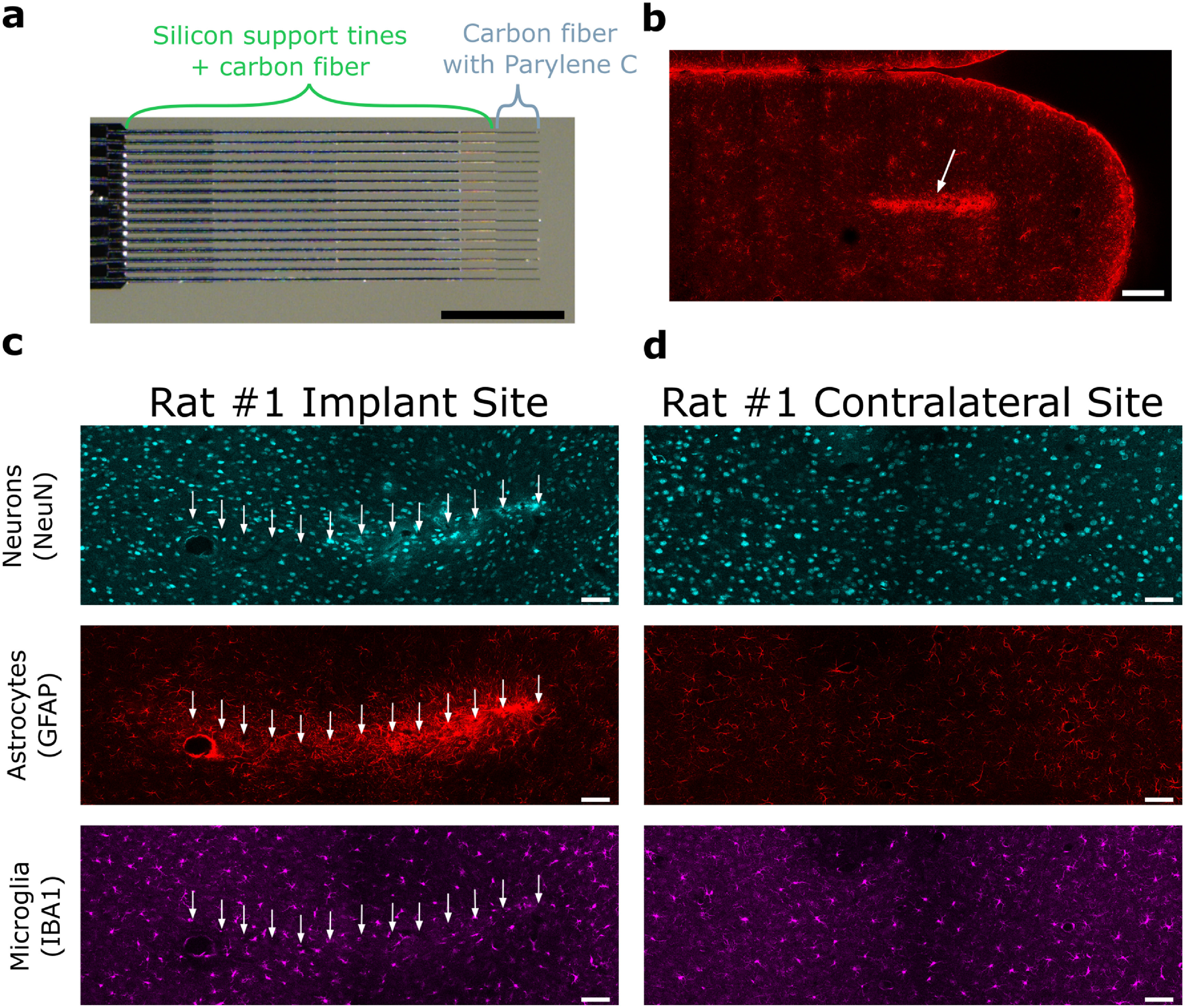
Electrode array identification and assessment of foreign body responses induced by whole carbon fiber electrode arrays implanted chronically. (a) Example of a HDCF electrode array implanted for this study. Tapering silicon support tines supported the fiber until the last 300 *µ*m of the fiber length, where the tip was the recording site. Scale bar, 1 mm. Image was slightly rotated in ImageJ. (b) Confocal image of astrocytes (GFAP) in the brain slice immediately dorsal to the brain slice with tips for rat #2. Glial responses dorsal to the tips from the support tines helped localize electrode tracts in ventral slices with tips. The arrow points to the implant site. Scale bar, 500 *µ*m. (c) Representative histology of the implant site in rat #1 (layer V motor cortex). Imaging plane was estimated to be up to 42 *µ*m ventral of some tips, and up to 12 *µ*m dorsal to others. (d) Representative histology of a site contralateral to the implant site for rat #1 with similar stereotaxic coordinates. Imaging settings were the same. (c and d) Scale bar, 80 *µ*m. (b)–(d), Cyan: NeuN (neurons); red: GFAP (astrocytes); magenta: IBA1 (microglia). White arrows point to fiber tracts identified in the tissue. Images were stitched (Preibisch *et al* 2009) and contrast adjusted in ImageJ. See also figure S3 for whole-array histology of rats #2 and #3.

We first verified that subcellular-scale (6.8 *µ*m diameter) carbon fiber electrode arrays implanted for this study yielded minimal FBRs similar to those observed with other carbon fiber electrode designs (Patel *et al* 2016, 2020, Welle *et al* 2020b). To assess the FBR along the arrays, we implanted one HDCF electrode array (Huan *et al* 2021) targeting layer V motor cortex in each of three rats for 12+ weeks. This design included tapering silicon support tines that extended up to the last 300 *µ*m of the fibers’ length to enable direct insertion (figure 1(a)) (Huan *et al* 2021). Similar to other implanted silicon electrodes (Turner *et al* 1999, Salatino *et al* 2017a), we found elevated GFAP staining around the silicon supports, indicating astrocyte enrichment as part of the FBR and signifying the implant site (figure 1(b)). The explanted electrodes left an approximately straight row of black holes in each image plane, illustrating the electrode tracts in 3D in brain slices containing the tips. At depths near putative electrode tips and far from the silicon supports (∼300 *µ*m), while enriched astrocytes persisted, minimal microglia responses and high neuron densities were observed (figure 1(c) and video 1). These microglial and neuronal responses appeared similar to those observed in previous reports (Patel *et al* 2016, 2020, Welle *et al* 2020b) and those located in symmetrical regions in the contralateral hemisphere (figure 1(d)). It is worth noting that we observed varying degrees of FBRs in histology showing the implant sites for all three rats (figures 1 and S3). Such variation may reflect differing structural damage during implantation (Ward *et al* 2009). Furthermore, we quantified glial fluorescent intensities with known carbon fiber locations for the first time. GFAP and IBA1 intensities were elevated up to 200 *µ*m and 60 *µ*m from putative electrode tips (figure S4), respectively, which is considerably closer than previous measurements with silicon shanks (Patel *et al* 2016).

### 3.2. Carbon fiber recording sites can be localized in post-explant motor cortex with cellular confidence

**Figure 2.**
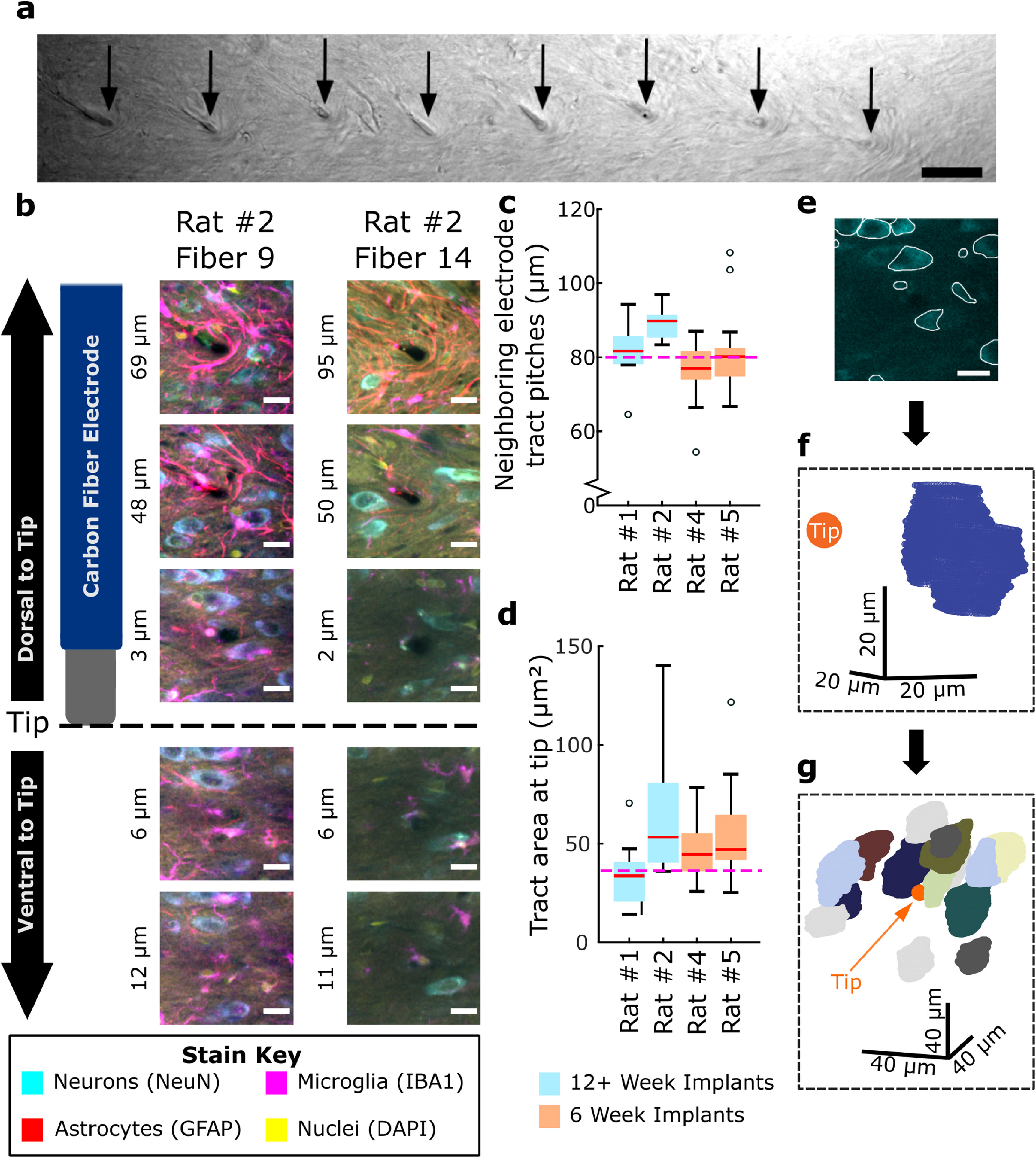
Carbon fiber recording sites can be localized in post-explant motor cortex with cellular confidence. (a) Transmitted light image of eight fiber tracts identified in rat #2. Black arrows point to dark holes where fibers had been implanted. Scale bar, 50 *µ*m. (b) Recording site tips of carbon fiber electrodes were identified by following the electrode tracts in the dorsal to ventral direction in 3D imaging volumes. Localization of two representative fibers is shown, with cartoon electrode accompanying (left). A black hole surrounded by glia or a disturbance in tissue, or both, is easily identified dorsal to the electrode tip (top). The tract was followed in the ventral direction until the hole was rapidly filled in by surrounding parenchyma and background fluorescence (images below dotted line). Scale bar, 20 *µ*m. Color scheme is the same as in figure 1, with yellow for cellular nuclei (DAPI) added. Images were contrast adjusted, histogram matched (Miura 2020) (twice for DAPI), and 3D Gaussian filtered (Ollion *et al* 2013) in ImageJ for easier visualization. (c) Electrode pitches measured for implanted fibers (*N* = 57 pitches). Dashed line is the expected value (80 *µ*m) from the design. (d), Cross-sectional area of fiber tracts at the tips (*N* = 61 fibers). Dashed line is the expected hole area for an uncoated fiber (36.3 *µ*m^2^). (c and d) Rats implanted for 12+ weeks shown in blue (*N* = 2), rats implanted for 6 weeks shown in orange (*N* = 2). (e)–(g), Quantification of neurons surrounding the recording site tips through neuron tracing. (e) Representative *z*-step (imaging plane) of neurons collected with confocal microscopy. The white outlines are traces that follow the edge of each neuron captured in the *z*-step imaging plane. Image is contrast adjusted, histogram matched, and median filtered. Scale bar, 20 *µ*m. (f) Tracing the neuron in each *z*-step that it was captured yielded a 3D scaffold of points (blue points). (h) Tracing neurons with centroids within 50 *µ*m from the recording site tip center yields an electrode-specific model of the surrounding neurons.

Localizing the recording sites of carbon fiber electrodes in histology can better inform the structure, function (Yang *et al* 2019), and health (Eles *et al* 2018) of nearby neurons that putatively contribute the spikes detected by the electrodes. In previous work, the recording site tips of carbon fibers could be localized in deep brain regions using a ‘slice-in-place’ method (Patel *et al* 2020). However, using this method to cryosection implants at superficial brain regions, such as motor cortex, is difficult due to the intrusion of supporting skull screws and the curvature of the brain. Here, we explanted the electrodes and estimated tip locations using biomarkers and visual cues captured with submicron-resolution confocal imaging instead. Tips could be identified by a combination of factors, including the FBR itself. Elevated GFAP immunostaining typically delimited electrode tracts as astrocytes enriched around a series of dark holes, particularly at shallower depths (figure 1(b)), but also at depths close to some tips. Simultaneous transmitted-light imaging demonstrated that these holes were not immunostaining artifacts (figure 2(a)). Such dark holes also appeared in other fluorescent channels contrasting with background staining. Successive confocal imaging produced high-resolution and high-contrast renditions in 3D. Collectively, we utilized the disappearance of these holes in the dorsal-ventral direction to corroborate fiber tracts and localize putative tips. For instance, as shown in figure 2(b) and videos 2 and 3, at the dorsal side of the brain slice, GFAP+ astrocytes wrapped around the dark hole of the electrode tract, signifying the fiber’s pre-explant location. This hole could then be followed in the ventral direction to a depth where it was rapidly filled in by surrounding parenchyma and background fluorescence, signifying the electrode tip.

We localized 29 putative tips of 31 fibers that were implanted for 12+ weeks (*N* = 2 rats), and 32 of 32 tips in an additional cohort implanted for 6 weeks (*N* = 2 rats). Two tips in rat #1 were not found, as their locations were likely included in a slice where some electrode tracts merged (data not shown). The distance between adjacent putative electrode tracts was measured at 82.1 ± 9.2 *µ*m, ($\bar{\text{X}}$ ± S, *N* = 57 pitches), which was close to the array design pitch (80 *µ*m) (figure 2(c)). The cross-sectional area of the tips was 50.5 ± 24.7 *µ*m^2^ (*N* = 61 fibers) and comparable to the expected 36.3 *µ*m^2^ from a bare carbon fiber (figure 2(d)). These measurements indicated that we correctly identified fiber locations. To estimate the precision in localizing tips, three observers independently corroborated tip locations (table 1). The median absolute difference between estimates was 9.6 *µ*m, suggesting subcellular precision. However, this difference was considerably lower in rats implanted for six weeks (5.2 *µ*m) than 12+ (14.7 *µ*m), likely the result of better tip positioning and elevated background staining. Furthermore, that the median difference in the horizontal plane was 2.2 *µ*m suggests that tip depth contributed more to tip localization error than tract position.

**Table 1.** Summary of comparisons of fiber tip localization by three reviewers. Each of three reviewers trained in histology localized putative carbon fiber tips and the absolute Euclidean distances between the localizations for each fiber were measured for comparison. Therefore, the number of comparisons was three multiplied by the number of fibers. Summary statistics were taken from the full samples of comparisons. The difference in the A/P and M/L plane was measured because images were collected in horizontally sliced brain sections, while the D/V axis was the axis of *z*-stack imaging. A/P: anterior/posterior. M/L: medial/lateral. D/V: dorsal/ventral.

Rat(s)	Number of fibers	Median absolute difference (*µ*m)	Interquartile range (*µ*m)	Median absolute difference in A/P & M/L (*µ*m)	Median absolute difference in D/V (*µ*m)
Rat #1 (12+ Weeks)	13	13.7	20.4	5.8	12.0
Rat #2 (12+ Weeks)	16	15.4	16.6	1.8	15.0
Rat #4 (6 Weeks)	16	6.9	9.2	1.4	6.6
Rat #5 (6 Weeks)	13 (3 excl.)	4.3	6.7	2.7	2.4
12+ Week Rats	29	14.7	17.5	2.9	13.8
6 Week Rats	29	5.2	9.0	1.8	4.8
Pooled	58	9.6	15.0	2.2	8.7

### 3.3. Neuron soma morphology is geometrically altered surrounding carbon fiber tips

**Figure 3.**
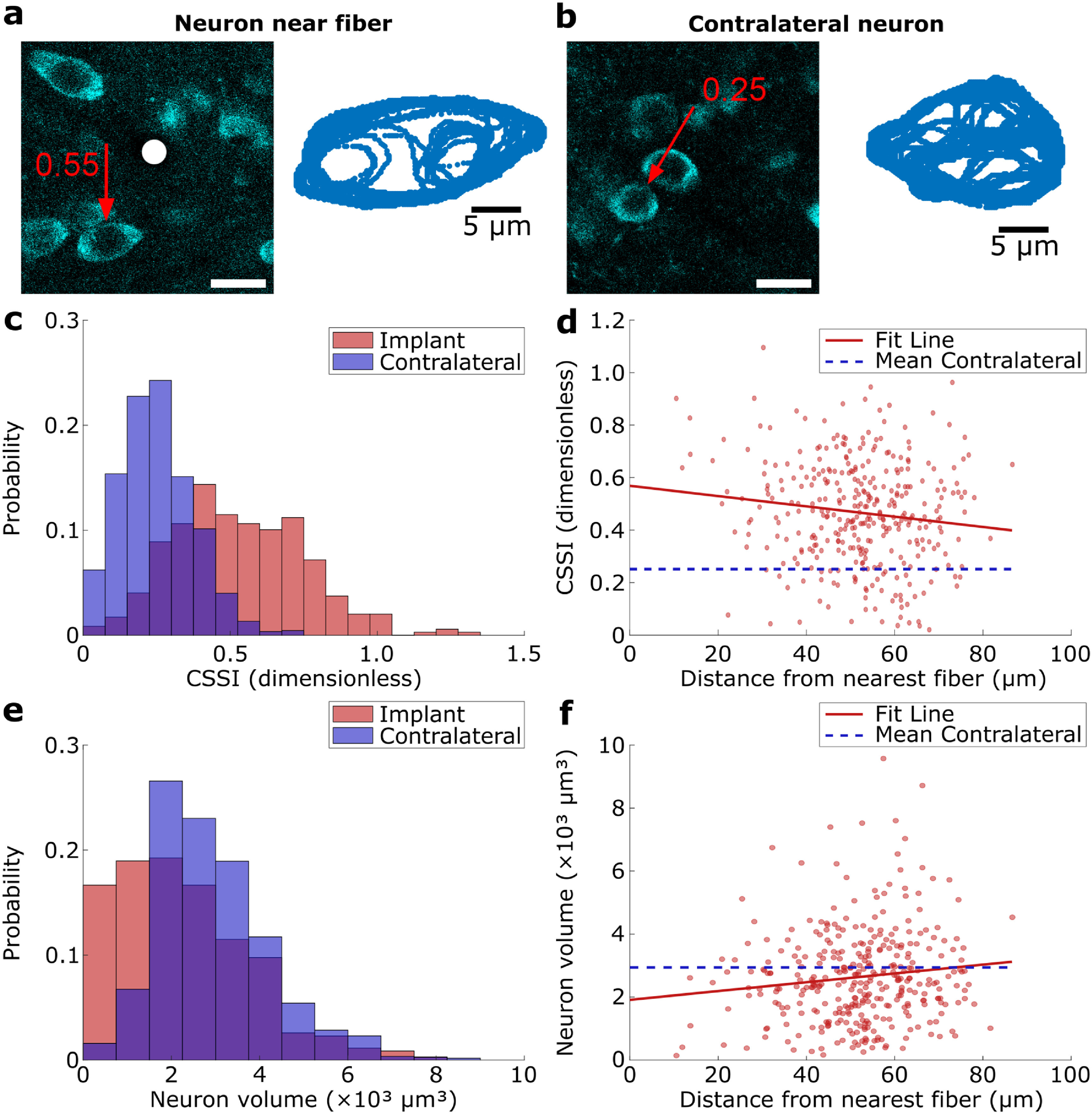
Neuron soma morphology is geometrically altered surrounding carbon fiber tips. (a) NeuN staining showing a representative neuron (CSSI (Du *et al* 2017) = 0.55) stretched by the presence of an implanted fiber (white dot) (left) and corresponding neural scaffold (right). Scale bar (left), 20 *µ*m. (b) Representative neuron in contralateral tissue (CSSI = 0.25) (left) and corresponding neural scaffold (right). This neuron has a lower CSSI and is more circular. Scale bar (left), 20 *µ*m. (c) Histograms showing the CSSIs calculated for neurons located within 50 *µ*m from recording site tips (N = 348 neurons) and neurons in contralateral tissue (*N* = 1125 neurons) without implants (*N* = 2 rats, 28 fibers). CSSIs around fibers were significantly larger (Kolmogorov–Smirnov test, *p* < 0.001). (d) CSSI vs. neuron distance (*N* = 354 neurons) from tip. Linear regression (solid line) yielded fit line *y* = −2.0 × 10^−3^ *x* + 0.57 (*R* = 0.13, *p* < 0.05). Mean CSSI (0.25) for contralateral neurons shown for comparison (dashed line). (e) Same as (c), but with neuron somal volumes (*N* = 348 neurons around fibers, *N* = 1125 neurons in contralateral). Distributions are significantly different (Kolmogorov–Smirnov test *p* < 0.001). (f) Same as (d), but with neuron somal volumes (*N* = 354 neurons). Linear regression (solid line) yielded fit line *y* = 14*x* + 1912 (*R* = 0.12, p < 0.05). Mean volume (2.9 × 10^3^ *µ*m^3^) for contralateral neurons is shown for comparison (dashed line).

Having localized the recording site tips with high confidence and captured nearby neurons with submicron resolution imaging, we sought to assess changes in neuron soma morphology near the implants, as previous studies reported that neurons surrounding silicon electrode implants were mechanically stretched compared to neurons in non-implanted regions (Du *et al* 2017, Eles *et al* 2018). We traced the 3D outlines of nearby neuron somas (*N* = 944 neurons, *N* = 28 fibers) and somas in symmetric contralateral sites (*N* = 1125 neurons) from NeuN staining images (figures 2(e)–(g) and S2) to measure soma shape and volume within a 50 *µ*m radial sphere from implanted fiber tips and hypothetical tips positioned in the contralateral hemisphere. Here, we also found that neurons close to the fiber tips appeared to be stretched in one direction compared to neurons in contralateral tissue (figures 3(a) and (b)). To quantify the degree of soma distortion, the CSSI was calculated using equation (1) (Du *et al* 2017, Eles *et al* 2018). The CSSI was defined as the ratio of the difference to the sum of the longest axis and the shortest axis of the soma, where a high CSSI indicates the soma has a more asymmetric shape and a CSSI near zero indicates a nearly circular soma (Du *et al* 2017). In figure 3(c), we plot the distributions of neuron soma CSSIs within a 50 *µ*m radius from implanted fibers (0.53 ± 0.22, $\bar{\text{X}}$ ± S, *N* = 348 neurons), and found those were 111% larger than those at the contralateral sites (0.25 ± 0.12, $\bar{\text{X}}$ ± S, *N* = 1125 neurons). Plotting the CSSI over the distance of each neuron relative to carbon fiber tips (red dots) allowed us to fit a linear regression line (red line) to extrapolate the distance at which neurons at the implant site would have CSSIs similar to the average CSSI (blue line) measured for neurons in the contralateral hemisphere (figure 3(d)). The slope of the linear regression (*R* = 0.13, *p* = 1.4 × 10^−2^) predicted this distance is 162 *µ*m. Since there was a morphological change for neurons in the close vicinity of the electrodes, we were also curious whether there was a concomitant change in neuron soma volume, which has not been investigated in previous FBR studies to our knowledge. We found that neuron somas within 50 *µ*m from fibers were 23% smaller (2.3 × 10^3^ ± 1.5 × 10^3^ *µ*m^3^, *N* = 348 neurons) than those in the contralateral hemisphere (2.9 × 10^3^ ± 1.3 × 10^3^ *µ*m^3^, *N* = 1125 neurons) (*p* < 0.001, Kolmogorov–Smirnov Test, figure 3(e)). Fitting soma volumes (red points) over distance to fibers resulted in a regression (red line, *R* = 0.12, *p* = 2.1 × 10^−2^) that predicted the neuron volume would increase to that of the contralateral hemisphere (blue line) at 74 *µ*m away from the fibers (figure 3(f)). We also compared neuron length in the dorsoventral direction along the direction of explantation to determine whether explanting the probes themselves may have influenced these morphological changes. While the distributions of neuron length were significantly different between the implant and control sites (*p* < 0.001, Kolmogorov–Smirnov Test), neurons at the implant site were 5.3% shorter on average and showed no meaningful trend over distance from the implant (*R* = 0.03, *p* = 0.55, linear regression) (figure S5).

### 3.4. Neuron placement around implanted carbon fibers resembles naturalistic neuron distributions

As we observed many neurons surrounding the arrays, we desired to quantify how naturally these neurons were distributed in the fibers’ immediate vicinities. Using the centroids of the aforementioned 3D reconstructions of neuron somas (figures 2(e)–(g)), we quantified neuron densities within a 50 *µ*m radial sphere from implanted and hypothetical control fibers. The neuron density around implanted tips was 3.5 × 10^4^ ± 0.9 × 10^4^ neurons mm^−3^ (*N* = 16 fibers), which was 82 ± 22% of that measured around hypothetical tips (*N* = 2932 fibers). In contrast, conventional single-shank silicon electrodes retain 40% of a healthy neuron density within 50 *µ*m (Winslow *et al* 2010) and 60% within 100 *µ*m (Biran *et al* 2005). As spikes from single neurons can putatively be separated into individual clusters within 50 *µ*m from an electrode (Henze *et al* 2000, Buzsáki 2004), the higher density around carbon fibers within this range may explain their improved recorded unit yield over silicon probes (Patel *et al* 2016).

**Figure 4.**
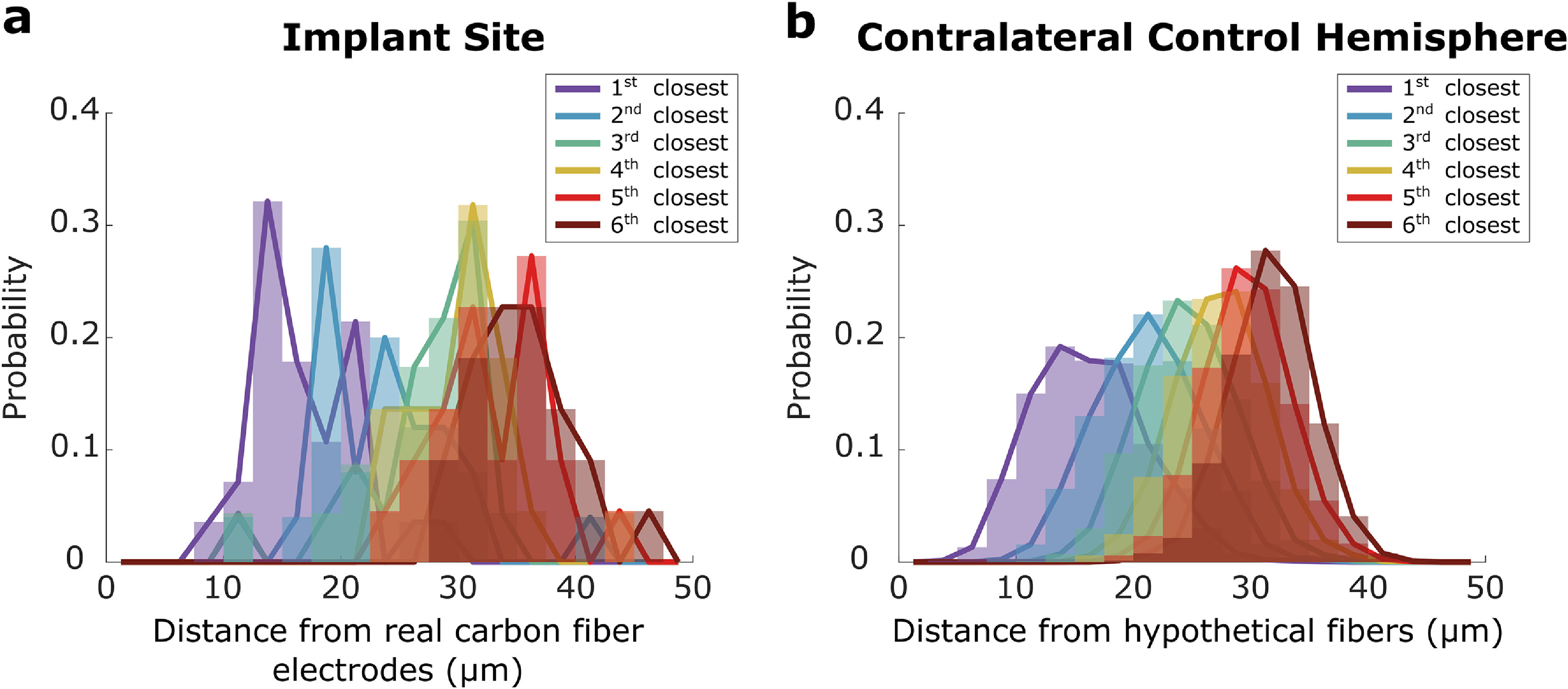
Neuron placement around implanted carbon fibers resembles naturalistic neuron distributions. (a) Histograms of the relative distances between the nearest six neurons and implanted carbon fiber electrodes in *N* = 2 rats. (b) Histograms of the relative distances between the nearest six neurons and points chosen as hypothetical fibers in the hemisphere contralateral to the implant sites in *N* = 2 rats. The contralateral sites had similar stereotaxic coordinates to the implant sites and were within the same brain sections. (a and b) see table 2 for a list of mean distances at each position.

Having confirmed that carbon fibers preserve most neurons within 50 *µ*m, we examined whether the nearest neuron positions relative to the tips were altered. This is important for modeling because the extracellular spike amplitudes recorded is inversely proportional to the distance between neurons and recording sites (Jog *et al* 2002, Lee *et al* 2007, Seymour *et al* 2017). To do so, we plot histograms of the distances between the nearest six somas’ centroids to each electrode tip (figure 4(a)) and compared them to the distances to each hypothetically positioned probe in the contralateral hemisphere (figure 4(b)). Importantly, the neuron nearest to tips was measured at 17.2 ± 4.6 *µ*m ($\bar{\text{X}}$ ± S, *N* = 28 fibers) away, which was only 1.0 *µ*m and not significantly farther away (*p* = 0.30, two sample *t*-test) than the neuron nearest to hypothetical tips in the contralateral hemisphere (16.2 ± 4.8 *µ*m, *N* = 2932 simulated fibers). We summarized the nearest six neuron positions in table 2. Of those six positions, the difference between neurons of matching positions around implanted and hypothetical fibers was of subcellular scale at 2.7 ± 1.0 *µ*m ($\bar{\text{X}}$ ± S). These results suggest that the neurons, which were mostly preserved with approximately proper positions, surrounding carbon fiber electrodes may have produced naturalistic physiological spiking activity.

**Table 2.** Nearest neuron positions surrounding carbon fibers. Closest six neuron positions relative to implanted carbon fiber electrodes (implant) and hypothetical fibers placed in contralateral tissue (contralateral). The number of fibers is different between positions because some fiber tips were too close to the top of the brain section to measure subsequent neuron positions (see Methods). The difference column shows difference in relative Euclidean distance between implanted fibers and hypothetical fibers in the contralateral hemisphere for each position.

Neuron position	Distance (Implant) $\bar{\text{X}}$ ± S *µ*m	Number of fibers	Distance (Contralateral) $\bar{\text{X}}$ ± S *µ*m	Number of simulated fibers	Difference (*µ*m)	Significance (*p* value)
1st	17.2 ± 4.6	28	16.2 ± 4.8	2932	1.0	0.30
2nd	23.3 ± 6.2	25	21.2 ± 4.5	2932	2.1	2.0 × 10^−2^
3rd	27.9 ± 6.0	23	24.7 ± 4.2	2932	3.3	1.9 × 10^−4^
4th	30.7 ± 4.6	22	27.4 ± 3.9	2932	3.3	9.8 × 10^−5^
5th	33.1 ± 4.8	22	29.6 ± 3.7	2932	3.5	1.6 × 10^−5^
6th	35.2 ± 4.4	22	31.7 ± 3.6	2932	3.5	6.6 × 10^−6^

### 3.5. Modeling suggests neuron distribution contributes to the number of sorted spike clusters

Previous work suggests that spikes can be sorted into clusters from individual neurons that were recorded as far as 50 *µ*m away from an electrode (Henze *et al* 2000, Buzsáki 2004). It has been assumed that each spike cluster includes action potentials from a single nearby neuron (Buzsáki 2004, Carlson and Carin 2019), but it is possible that the electrode records similar spike waveforms generated from multiple neurons (Lewicki 1998). At the same time, it has been widely noted that the number of clusters determined by spike sorting, reported to be between one and four (Rey *et al* 2015) and between one and six clusters (Buzsáki 2004, Shoham *et al* 2006, Pedreira *et al* 2012), is at least an order of magnitude lower than the neuron count within a 50 *µ*m radius in mammalian brain (Buzsáki 2004, Shoham *et al* 2006, Pedreira *et al* 2012). The FBR is speculated to contribute to this discrepancy (Pedreira *et al* 2012), as fewer viable neurons are observed surrounding electrodes after chronic implantation (Polikov *et al* 2005). Particularly salient is a 60% percent reduction within 50 *µ*m (Winslow *et al* 2010). Other possible factors are that neurons remain non-active during recording sessions (Shoham *et al* 2006, Pedreira *et al* 2012) or spike-sorting methods are currently insufficient (Pedreira *et al* 2012, Carlson and Carin 2019). Presently, this discrepancy remains unresolved.

**Figure 5.**
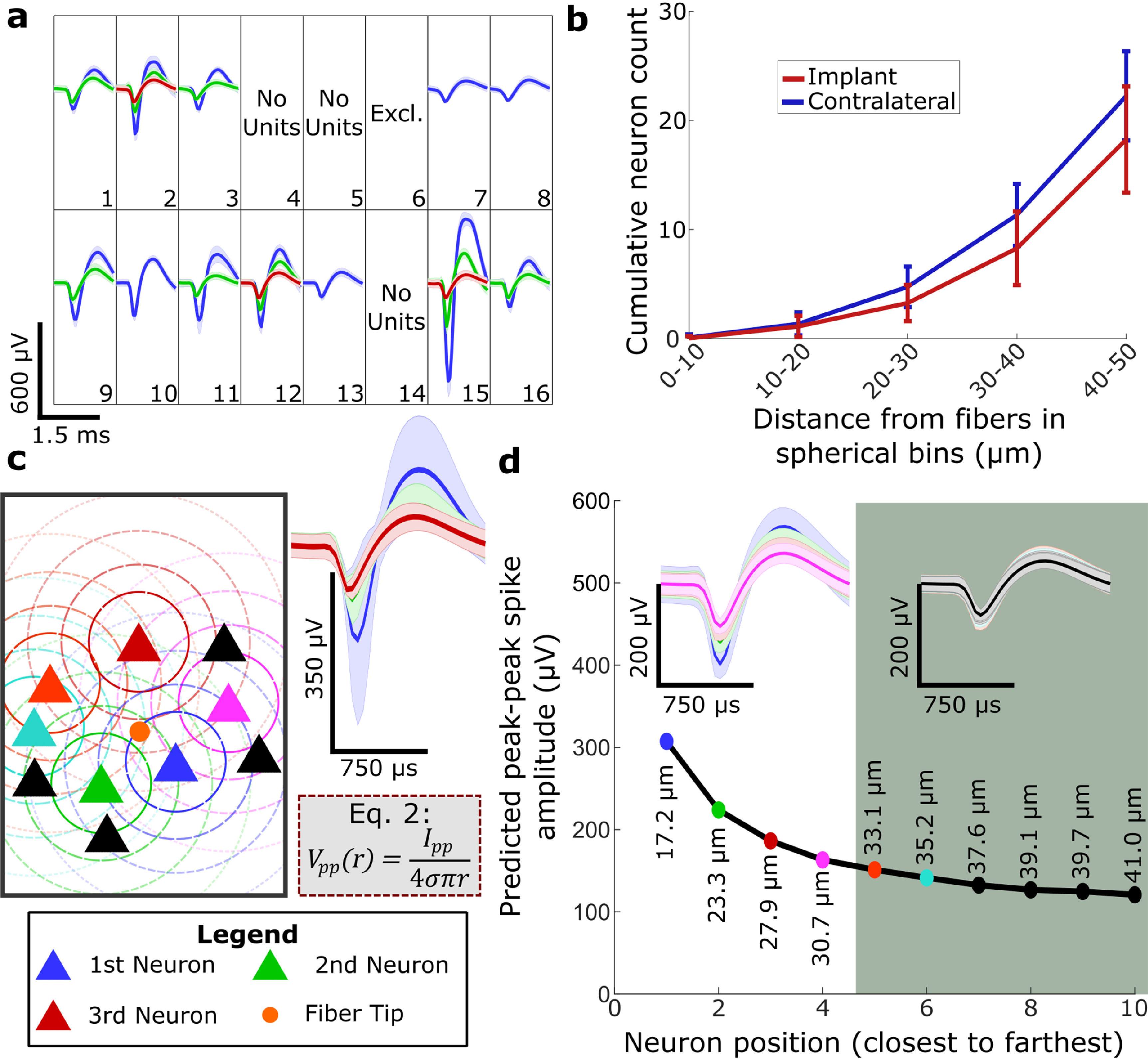
Modeling suggests neuron distribution contributes to the number of sorted spike clusters. (a) Spike sorted electrophysiology from a recording session that yielded exemplary units. Recorded on day 84 of implantation for rat #2. Solid lines show mean cluster waveform and shading shows cluster standard deviation. Channels marked ‘Excl.’ were excluded because of high 10 Hz impedance (see *Electrophysiological recording and spike sorting* in *Methods*). (b) Cumulative neuron count at each 10 *µ*m spherical bin from implanted carbon fiber electrode tips (red). Cumulative neuron counts at each bin surrounding simulated fibers in contralateral cortex shown in blue. Error bars show standard deviation. (c) Pictorial representation of the simple point source model described by equation (2). The carbon fiber electrode tip (orange dot) and neurons (triangles) are treated as point sources. The nearest three neurons (blue, green, and red triangles, in order) produce the three largest units (right), which were determined from all spikes attributed to the largest three units from the spike panel in (a). Neuron positions in this graphic are approximately proportional to relative distances measured in histology. Equation (2) is reproduced below the spikes. (d) Predicted mean peak–peak amplitude for the closest ten neuron positions relative to implanted carbon fibers as observed from histology, where the color of each dot corresponds to the neuron of the same position in (c). Neuron positions are in order from closest to farthest. Mean spike waveforms matching mean peak–peak amplitude are displayed above the curve and match each corresponding point by color. Waveforms are divided into two zones, the ‘easy sorting zone’ (left, white background), where neighboring neuron spike amplitudes differ by >15 *µV*_pp_, and the ‘hard sorting zone’ (right, grey background) where neighboring neuron spike amplitudes differ by <15 *µV*_pp_. See also figure S8 for all waveforms grouped into one plot. Shading shows standard deviation.

Since carbon fiber electrodes maintained a nearly natural distribution of the nearest six neuron positions, which were within 50 *µ*m from the recording sites, we were well positioned to further consider this discrepancy by modeling electrophysiology. Also, since carbon fiber electrodes yield higher SNRs than silicon electrodes (Patel *et al* 2016) and higher SNRs are expected to yield more spikes (Carlson and Carin 2019) with higher accuracy (Magland *et al* 2020), we anticipated spike sorting more clusters. Therefore, we sorted electrophysiology from an exemplary recording session towards the end of the implantation period (day 84 of 92) that yielded large spike clusters, where the mean peak–peak amplitude of the largest cluster recorded on channels that yielded clusters was 354.8 ± 237.2 *µV*_pp_ ($\bar{\text{X}}$ ± S, *N* = 12 channels) and the single largest cluster had a mean amplitude of 998.9 *µV*_pp_ (figure 5(a)). Furthermore, 12 of 16 channels yielded spike clusters with mean amplitude >100 *µV*_pp_, signifying a high chronic signal yield consistent with previous work (Welle *et al* 2020b). Signal yield at experimental endpoints for all five implanted devices are presented in figure S1. However, our spike sorting for this exemplary recording session yielded a median of 2 and maximum of 3 spike clusters per electrode, which is considerably lower than the 18.3 ± 4.9 neurons observed within 50 *µ*m (*N* = 16 fibers) even after inducing a lower FBR with high signal yield.

We hypothesized that neuron distribution itself may contribute to the low number of sorted clusters. As expected (Henze *et al* 2000) from the geometry of concentric spheres with increasing radius, the number of neurons relative to the electrode grew rapidly with increasing spherical volume. On average, we found fewer than one neuron (0.0 ± 0.2) within 10 *µ*m, three neurons (3.3 ± 1.7) within 30 *µ*m, and fifteen neurons (15.3 ± 4.1) 30–50 *µ*m away from fiber tips (figure 5(b)). Given the inverse relationship between neuron distance and the recorded spike amplitude (Jog *et al* 2002, Buzsáki 2004, Pedreira *et al* 2012), the large neuron count at further distances would likely generate similar spiking amplitudes that would be difficult to sort (Pedreira *et al* 2012).

We used the simplest point source model where neurons are treated as points (Lee *et al* 2007, Nason *et al* 2020) and each neuron has the same spiking output (Lempka *et al* 2011). In this paper, a point source is defined as the centroid of a neuron’s NeuN stain. Figure 5(c) illustrates this model with the relative distances of the closest ten neurons based on our measurements (table 2). We fit the model for *I*_pp_ using spikes sorted into the largest three units’ mean spike amplitudes (*N* = 12 channels) (figure 5(c)) and the nearest three neurons’ mean positions (table 2), where conductance, *σ,* was 0.27 S m^−1^ (Slutzky *et al* 2010). This fit yielded *I*_pp_ = 16.6 nA (figure S6(a)), which is similar to the 10 nA determined from fitting 60 *µ*V to 50 *µ*m, as recommended by Pedreira *et al* (2012) from combined intracellular and extracellular recordings (Henze *et al* 2000, Buzsáki 2004). To verify this parameter, we fit the model in two other ways by matching each sorted cluster with a respective neuron positioned around the same electrode (e.g. the second largest cluster associated with the second closest neuron) (figure S6(b)). Fitting for both *I*_pp_ and *σ* yielded similar values of 14.4 nA and 0.23 S m^−1^, respectively, while fitting for just *I*_pp_ yielded *I*_pp_ = 15.9 nA. That all three modeling approaches produced similar results (figure S6) that were consistent with literature suggests this model can reasonably predict spike amplitudes with neuron distance as input.

Using their average relative positions to implanted fibers in the model, we plot the average spike amplitudes of the nearest ten neurons to determine spike cluster discriminability (figure 5(d)). As expected from the neuron distribution we observed, predicted amplitudes quickly approached an asymptote as neuron position increased. Since baseline noise may contribute to spike detection and cluster differentiability (Du *et al* 2009, Pedreira *et al* 2012), we hypothesized that noise may obfuscate these small differences in amplitudes from neighboring neurons. Previous work comparing noise levels recorded in Michigan-style silicon probes and carbon fiber electrodes probes measured baseline noise levels of 10 and 15 *µV*_rms_, respectively (Patel *et al* 2016). Therefore, we compared the average difference in spiking amplitude between consecutive neurons to these noise levels to estimate neuron differentiability (figure S7). This difference remained smaller than 15 *µV*_pp_ for comparisons of neuron positions beyond the third and fourth neuron and smaller than 10 *µV*_pp_ for comparisons beyond the fourth and fifth neuron. Therefore, the fourth closest neuron (30.7 ± 4.6 *µ*m) is situated along the boundary at which spike clusters become indistinguishable, capturing activity from multiple neurons, and is consistent with the 1–4 sortable clusters typically observed in literature (Rey *et al* 2015). To further illustrate, we grouped simulated spikes according to differentiability, where the four largest clusters are more easily separable than the fifth through the tenth (figure 5(d)) and plotted all ten waveforms together (figure S8). When separated, the four largest units could easily merge into two or three units with variation in position and consequently amplitude. This is plausible given that within the four closest neurons observed for 11/28 fibers, at least two neighboring neuron positions were within 1 *µ*m apart. Similarly, when plotted together, the clusters begin to merge starting with the fourth largest cluster.

## 4. Discussion

Here, we consistently localized the positions of carbon fiber electrode tips that had been chronically implanted in motor cortex with subcellular-scale confidence for the first time. Since our confidence measurement was based upon independent measurements made by three individuals, we estimate that this measurement is also evidence of our method’s reproducibility. Volumetric confocal imaging of the implant site enabled this localization and an examination of the surrounding neurons and FBR with more 3D details and precision (Biran *et al* 2005, Nolta *et al* 2015, Patel *et al* 2016, 2020, Black *et al* 2018, Yang *et al* 2019, Welle *et al* 2020b, Sharon *et al* 2021). In particular, we focused on the nearest 50 *µ*m regarded as the single-unit recording zone (Henze *et al* 2000, Buzsáki 2004), in which we observed a neuron distribution similar to that of healthy cortex. In contrast, multi-shank silicon arrays such as the UEA can remodel the nearby neuron distribution by inducing large tissue voids (Nolta *et al* 2015, Black *et al* 2018) and widespread necrosis (Szymanski *et al* 2021). A more direct comparison can be made with Michigan-style electrodes, which can induce a 60% neuron loss within the 50 *µ*m recording radius (Winslow *et al* 2010). Given that we observed an 18% neuron loss on average within this radius of carbon fibers, a conservative estimate suggests that carbon fibers retain at least nine more neurons per penetrating electrode on average without accounting for the shanks’ larger sizes. When scaling up to the 100+ channels used in BMI devices, several hundreds of neurons could be preserved by carbon fibers instead of silicon shanks. Carbon fibers have also proven to have much higher functional probe yield and record more units per probe with better recording SNR (Patel *et al* 2016, Black *et al* 2018, Welle *et al* 2020b). Therefore, our work provides strong evidence that subcellular-scale electrodes such as carbon fibers retain recordable neurons to a considerably greater degree.

At the same time, our detailed examination revealed components of the FBR that could inform future designs. This included neuron soma stretching around carbon fibers, which has similarly been observed surrounding conventional microwire and silicon electrodes and has been attributed to chronic micromotion relative to surrounding tissue (Du *et al* 2017) or to electrode insertion during surgery (Eles *et al* 2018). We considered whether the removal of the electrode arrays post-fixation may have contributed to these morphological changes by determining whether neurons were stretched along the direction of explantation, as the removal likely generated considerable strain in the immediate vicinity of the probes. Given that there was no meaningful difference in neuron length in that direction and that neurons have previously been shown to stretch along the horizontal plane surrounding soft (Du *et al* 2017) and carbon fiber electrodes (Patel *et al* 2020) that were sliced in place, these morphological changes must have occurred prior to euthanasia. Also, the persistence of astrocyte enrichment along the electrode tracts to depths close to the tips suggests that the array architecture may induce a greater FBR than previous carbon fiber designs, which have shown a minimal response (Patel *et al* 2016, 2020). That the astrocyte response reduces closer to the putative tips suggests that the permanent silicon shuttles, even with small feature size (15.5–40.5 *µ*m), may have been the foremost contributor to this increased FBR. As sharpening the tips of electrodes with microwire-like geometry enables facile and unsupported insertion to deep cortical depths, such as 1.2 mm (Welle *et al* 2021) and 1.5 mm (Obaid *et al* 2020b) deep, future iterations could use fire-sharpening (Guitchounts *et al* 2013, Welle *et al* 2021) or electro-sharpening (El-Giar and Wipf 2006, Obaid and Wu *et al* 2020b, Sahasrabuddhe *et al* 2021) to obviate the need for shuttles and to reduce insertion force (Obaid and Wu *et al* 2020b). Additionally, although Massey *et al* (2019) reported a higher carbon fiber electrode pitch at 38 *µ*m, the pitch reported here is the highest that has been assessed with histology after a chronic implant at 80 *µ*m. This higher pitch may have been a factor in the increased FBR that we observed, and warrants a histological sensitivity analysis of the effect that microwire or microwire-like shank density may have on the surrounding tissue. Furthermore, skull-fixing the electrodes induces an increased FBR compared to floating electrodes (Biran *et al* 2007). Given that the neuron distortion observed here likely accompanied implant micromotion (Du *et al* 2017), subcellular-scale electrode designs should adopt a floating architecture similar to that of the UEA, as flexible electrodes such as syringe-injectable (Hong *et al* 2015, Schuhmann Jr. *et al* 2017, Yang *et al* 2019) and NET (Luan *et al* 2017) probes already have, even with the added benefits of their small size. At the same time, our close inspection of the immediate vicinity of the tips with known positions may have uncovered a previously overlooked and increase in the FBR that is more pronounced at the recording site tips. This is plausible as a moderate increase in GFAP and IBA1 intensity was observed previously surrounding carbon fibers compared to unimplanted tissue (Kozai *et al* 2012).

Having visualized an approximately naturalistic neuron distribution around carbon fibers and recorded electrophysiology from them, we sought to characterize the relationship between the surrounding neuron population and spikes recorded through modeling. We considered predicting individual neuron positions with electrophysiology, but tip localization would need to be more precise. Although our estimated localization error was lower (9.6 *µ*m, table 1, <1 soma) than the approximate error of 3–4 somata reported in recent work by Marques-Smith *et al* (2020), which attempted neuron localization using Neuropixels probes (Jun *et al* 2017), our error was higher than the average difference in position between the first and second closest neurons (6.1 ± 5.1 *µ*m) and subsequent positions (figure S9). Additionally, our point source model was simplistic, and likely would have to incorporate more biological phenomena to accurately predict the locations of surrounding neurons. Previous modeling and *in vitro* recordings suggest that dendritic morphology (Pettersen and Einevoll 2008) and the axon initial segment (Bakkum *et al* 2019) contribute greatly to the extracellular potentials recorded, respectively, and therefore immunostaining and 3D segmenting these structures in addition to neuron somata (NeuN) and accounting for their contributions to recorded potentials may increase the accuracy of our model. Previous work has shown that *in vivo* optical imaging, such as two-photon imaging, can be used to measure the morphological changes of nearby genetically-labeled brain cells (Eles *et al* 2018) and other elements of the FBR over the course of chronic implantations surrounding non-functional probes (Kozai *et al* 2010, 2016, Wellman *et al* 2019, Savya *et al* 2022) and visualize neuronal firing *in situ* (Lin and Schnitzer 2016). However, our functional study required the installation of the whole recording headstage and connector, which would block the cranial window for optical imaging. Additionally, optical imaging is limited to shallow cortical layers (Siegle *et al* 2021), which is not suitable for deeper brain regions, such as the depth to which we inserted the carbon fibers to record layer 5 motor neurons in the rat brain. In summary, despite these limitations, our method provides a viable solution for assessment of the FBR at the end point for similar recording devices, including the Utah array, and modeling recorded electrophysiology at the level of the neural population.

Regardless, modeling the ten closest neurons’ spike amplitudes suggests that the fourth closest may be situated on the boundary at which clusters become indistinguishable and may explain the low number of clusters (1–4) (Pedreira *et al* 2012, Rey *et al* 2015) attributed to individual neurons. That this single unit boundary is 30.7 ± 4.6 *µ*m away and dependent on neuron distribution disagrees with the notion that this boundary is 50 *µ*m (Henze *et al* 2000, Buzsáki 2004). That said, spikes from neurons up to 140 *µ*m away have been reported as distinguishable from background noise in the hippocampus, thereby contributing to multi-units beyond the above-mentioned single unit boundary (Henze *et al* 2000, Buzsáki 2004). However, this distance may also be brain region dependent, because recording in regions with varied neuron densities or cell type distributions (Collins *et al* 2010, Herculano-Houzel *et al* 2013) likely produce distinct background noise levels (Lempka *et al* 2011) that heavily influence spike detectability. Therefore, the neuron density of target regions must be considered when interpreting intracortical electrophysiology and should influence electrode design parameters such as recording site pitch (Kleinfeld *et al* 2019). Additionally, that our exemplary recording session yielded a median of two clusters suggests that other previously identified factors, such as silent neurons (Shoham *et al* 2006, Pedreira *et al* 2012) (although disputed by Marques-Smith *et al* (2020)), limitations in spike sorting algorithms (Pedreira *et al* 2012), and baseline noise (Du *et al* 2009) may have contributed to the low cluster count. Furthermore, recent work suggests that favorable histological outcomes may not correlate with high recording yield (Michelson *et al* 2018). This may be explained by neuronal hypoexcitability following electrode implantation (Eles *et al* 2018) or an observed shift toward a higher proportion of activity from inhibitory neurons surrounding chronic implants (Salatino *et al* 2017b, Michelson *et al* 2018). Therefore, our results contribute to an increasing list of factors that comprise the mismatch between cluster sortability and histology. Combined with recent work demonstrating that the downstream biological effects of electrode implantation are more complex than traditionally thought, our modeling and assessments of the FBR corroborate the need for further investigation into the interactions between electrodes and surrounding tissue, even for designs more biocompatible than traditional silicon electrodes (Salatino *et al* 2017a, Michelson *et al* 2018, Thompson *et al* 2020, 2021).

## 5. Conclusion

In this work, we demonstrated that the recording site tips of subcellular-scale carbon fiber electrodes can be localized with cellular-subcellular resolution after explanting the electrodes. This enabled measurement of the surrounding neurons in 3D, which indicated that their somata were stressed, but were still positioned in a nearly natural distribution. Modeling the electrophysiological signals that this geometric distribution of neurons might produce suggests that the low number of spike clusters typically identified in spike sorting may arise, at least partially, in neuron placement, and likely varies with neuron density. Overall, our work informs design considerations for carbon fiber electrodes and other intracortical electrodes with similar subcellular feature size.

## Data Availability

The data that support the findings of this study are available upon reasonable request from the authors.
